# Transcriptomic analysis reveals the dynamic changes of transcription factors during early development of chicken embryo

**DOI:** 10.1186/s12864-022-09054-x

**Published:** 2022-12-13

**Authors:** Liqin Liao, Ziqi Yao, Jie Kong, Xinheng Zhang, Hongxin Li, Weiguo Chen, Qingmei Xie

**Affiliations:** 1 Heyuan Branch, Guangdong Provincial Laboratory of Lingnan Modern Agricultural Science and Technology, College of Animal Science, South China Agricultural University, Guangzhou, 510642 China; 2 Guangdong Provincial Key Lab of Agro Animal Genomics and Molecular Breeding, Guangzhou, 510642 China; 3 South China Collaborative Innovation Center for Poultry Disease Control and Product Safety, Guangzhou, 510642 P. R. China; 4 Key Laboratory of Animal Health Aquaculture and Environmental Control, Guangzhou, Guangdong 510642 P. R. China

**Keywords:** Embryogenesis, Chicken, Transcription factors, Gene expression, Network analysis

## Abstract

**Background:**

The transition from fertilized egg to embryo in chicken requires activation of hundreds of genes that were mostly inactivated before fertilization, which is accompanied with various biological processes. Undoubtedly, transcription factors (TFs) play important roles in regulating the changes in gene expression pattern observed at early development. However, the contribution of TFs during early embryo development of chicken still remains largely unknown that need to be investigated. Therefore, an understanding of the development of vertebrates would be greatly facilitated by study of the dynamic changes in transcription factors during early chicken embryo.

**Results:**

In the current study, we selected five early developmental stages in White Leghorn chicken, *gallus gallus*, for transcriptome analysis, cover 17,478 genes with about 807 million clean reads of RNA-sequencing. We have compared global gene expression patterns of consecutive stages and noted the differences. Comparative analysis of differentially expressed TFs (FDR < 0.05) profiles between neighboring developmental timepoints revealed significantly enriched biological categories associated with differentiation, development and morphogenesis. We also found that Zf-C2H2, Homeobox and bHLH were three dominant transcription factor families that appeared in early embryogenesis. More importantly, a TFs co-expression network was constructed and 16 critical TFs were identified.

**Conclusion:**

Our findings provide a comprehensive regulatory framework of TFs in chicken early embryo, revealing new insights into alterations of chicken embryonic TF expression and broadening better understanding of TF function in chicken embryogenesis.

**Supplementary Information:**

The online version contains supplementary material available at 10.1186/s12864-022-09054-x.

## Introduction

Transcription factors (TFs) interpret the genome directly, and are responsible for decoding DNA sequences [1]. It is reported that transcriptional factors are key components of cells that control gene expression, determining how the cells function [2]. Transcription factors acting as conductor orchestrate complex regulatory networks of gene expression. A deeper understanding of the common transcription factors and their shared interaction by analyzing a set of coregulated or differentially expressed genes can provide insight into the pathways underlying such expression patterns [3]. Embryonic development involves a mass of cells achieving specific cell identities depending on morphogen gradients and the activation of transcription factors (TFs) [4]. Embryos in the early stages of their development show transcriptional activities that are different from those occurring later. Normally, changes in the gene expression are regulated by transcription factors, which play crucial roles in biological processes such as cell proliferation, cell differentiation.

Successful embryo development is dependent on the early stages of embryogenesis and the proper activation of the genome. For example, T-box factors are an ancient family of transcription factors that govern gene expression patterns that are critical for embryonic development [5], such as Tbx5 and Tbx4 binding with LMP-4 with important roles in vertebrate limb and heart development [6]. The transcription factors fork-head box (Fox) is commonly conserved in organisms varying from yeast to humans [7]. In the chicken reproduction development, Fox family is a prominent regulator for development of testis or ovarian [8, 9]. Moreover, it is considered critical to identify regulatory elements within the promoter region in order to understand the mechanism underlying transcriptional regulation in specific cell types [10], such as Sox11 activating Prox1 expression through multiple regulatory elements to promote chicken embryonic neurogenesis [11], transcription factor Sox2 binding with Cped1 to regulate the formation of chicken spermatogonial stem cells [12].

Chicken is one of the most important commercial species as well as a model organism for biological and medical research (chicken genomics). An increasingly efforts to character transcripts in chicken by RNA-sequencing have provided key insights into function of the chicken genome, such as the transcriptome analysis of early embryo to distinct gene clusters with specific morphological changes [13], revealing the chicken specific signaling pathways and gradually analogous gene expression via zygotic genome activation (ZGA) by RNA-sequencing [14], as well as analysis of transcriptome-wide m6A methylation modification pattern in the gonads of chicken embryos [15]. The study of embryogenesis is critical for a comprehensive understanding of the gene expression patterns and underlying biological changes during early embryonic developmental stages of an organism. The transcriptome profiling of chicken embryos creates an opportunity to advance our understanding of the molecular regulation of embryo development. Nevertheless, researches about transcription factors in chicken genome mainly focus on studying functions of specific factors, such as: 1) the fact that chicken NANOG, SOX2, and POUV expression varies dramatically at different stages shows that chickens have a distinctive pluripotent circuitry and may be crucial in the early development of pluripotency; 2) Chicken C/EBP has the ability to directly bind to and activate the PPAR gene promoter, which is one of the primary controllers of adipogenesis [16, 17]. However, the whole transcription factors landscape of early chicken embryo remains unclear. Here, we focused on early chicken embryo development underlying its diverse transcription factors and investigated the distribution and expression pattern of TFs.

In this study, we used RNA-sequencing to systematically investigate the expression profiles of all annotated transcription factors of chicken during early development stages. Five early developmental stages, including 1, 2, 3, 4 and 5 days after fertilization, were selected for transcriptome sequencing and analysis. We have identified differentially expressed genes (DEGs) between neighboring developmental stages. Identifying key genes and pathways involved in the regulation of embryonic development was achieved by analyzing differentially expressed transcription factors (DE-TFs) across five stages of development. The DE-TFs were used to conduct Gene Ontology (GO) enrichment analysis to reveal the biological functions. Importantly, this is the first comprehensive regulatory framework for transcription factors in early embryogenesis in chickens, highlighting the dynamics of TFs expression at the early stages of embryo.

## Materials and methods

### Ethics statement

All of the experimental protocols involved in animal care and sample collection were approved by the Animal Ethics Committee at the South China Agricultural University, China (approval ID: SYXK-2022-0136).

### Embryos collection and RNA extraction

Fertilized eggs from *White Leghorns* were purchased from Guangdong Wen’s DaHuaNong Biotechnology Co., Ltd. The eggs were incubated at 37.5 °C and 65% relative humidity in an automated egg incubator, rotating every 6 h. Embryos were collected at the following times point: 24 h, 48 h, 72 h, 96 h, and 120 h, with three biological replicates for each embryonic stage, labeled Em1d-Em5d. Total RNA was extracted using Trizol reagent kit (Invitrogen, Carlsbad, CA, USA) according to the manufacturer’s protocol. The RNA concentration and purity were measured using the Nano-Drop 2000 spectrophotometer (Thermo Fisher Scientific, Wilmington, DE, USA). RNA quality was assessed on an Agilent 2100 Bioanalyzer (Agilent Technologies, Palo Alto, CA, USA).

### Library construction and sequencing

Constructing cDNA library was performed as previous studies following the instructions of the manufacturer provided by the GENE-DENOVO Biotechnology [18–20]. Briefly, after total RNA was extracted, eukaryotic mRNA was enriched by Oligo(dT) beads, while prokaryotic mRNA was depleted by removing rRNA by Ribo-Zero™ Magnetic Kit (Epicentre, Madison, WI, USA). Then the enriched mRNA was fragmented into short fragments using fragmentation buffer and was reverse transcribed into cDNA with random primers. Second-strand cDNA were synthesized by DNA polymerase I, RNase H, dNTP and buffer. Then the cDNA fragments were purified with QiaQuick PCR extraction kit (Qiagen, Venlo, The Netherlands), end repaired, A base added, and ligated to Illumina sequencing adapters. The ligation products were size selected by agarose gel electrophoresis, PCR amplified, and sequenced using Illumina Novaseq6000. Library construction and sequencing reactions were conducted at GENE-DENOVO Biotechnology Co., Ltd (Guangzhou, China). The raw RNA-seq data is available at NCBI (PRJNA850787).

### Transcriptome assembly

Reads were further filtered according to the following rules to obtain high-quality clean reads by fastp (version 0.18.0). Firstly, deleting adapter-containing reads; secondly, readings with more than 10% unknown nucleotides (N) are also removed; thirdly, we removed all reads with terminal poly A; lastly, eliminating low quality reads (containing more than 50% number of bases with mass value Q ≤ 20). The short reads alignment tool Bowtie2 was used to compare the clean reads to the ribosome database of the species [21]. After comparative analysis based on the chicken genome (GRCg6a) using the HISAT2 software [22], we re-constructed the transcriptome by StringTie and then counted the expression of each gene via RSEM [23, 24].

### Gene expression analysis

Gene expression was presented with fragments per kilobase of transcript per million fragments mapped (FPKM). Principal component analysis was used to assess sample repeatability. The DESeq2 tool was used to perform differential expression analysis between the five stages. Genes with FDR (false discovery rate) ≤ 0.05 and Fold Change ≥ 2 were considered as DEGs between two stages. Simultaneously, the ggplot2 software was used to carry out a hierarchical cluster analysis of differentially expressed genes. (http://www.r-project.org/). The final lists of unique genes were used for further analysis after duplicate and missing values were removed.

### Detection of TFs in the list of DEGs

To identify the TFs that have differentially expression levels as they go from one stage to the next, we performed Hidden Markov Model scan (hmmscan) to compared the lists of DEGs with the Animal Transcription Factor DataBase [25]. Raw data for DEGs and DE-TFs can be found in the supplementary files.

### Network construction and analysis

All DE-TFs and their target genes were applied to construct the co-expressed network. Protein–protein networks were constructed by extracting the information regarding TFs interactions from STRING database [26]. Cytoscape [27] software were used to visualize and analyze the networks. Moreover, hub TFs were analyzed by KEGG and shown by Sankey plot.

### Functional annotation of TFs

The Gene Ontology (GO, http://www.geneontology.org/) terms for biological process, cellular component, and molecular function categories [28], as well as Kyoto Encyclopedia of Genes and Genomes (KEGG) pathways (https://www.kegg.jp/kegg/) [29–31], were enriched based on the OmicShare online tool with default parameters (https://www.omicshare.com/). *P*-value < 0.05 were considered to be significantly enriched.

### Data validation by quantitative real-time PCR

Embryonic gene expression analysis for 16 selected hub TFs, based on RNA-seq results, was validated by Quantitative real-time polymerase chain reaction (qRT-PCR). qRT-PCR was performed with an CFX96™ Real-Time system (BIO-RAD, USA) using the SYBR Green qPCR Master Mix (Bimake, China) according to the manufacturer’s instructions. The primers were designed by Primer Premier5 software. GAPDH was used as the internal reference, and the sequences of the gene-specific primers are listed in Table 1. The comparative Ct method (2^−△△Ct^ method) was used to calculate the relative gene expressions of the samples, which were normalized using the GAPDH mRNA level.

**Table 1:** List of primer sequences used in qRT-PCR

Gene	Primer		Accession No.	Product length (bp)	Annealing temperature (℃)
*POU5F3*	F: GGAGCAGTTTGCCAAGGA R: GAAGCGGCAGATGGTGGT		NM_001309372.2	124	61
*NANOG*	F: CAGGTGAAGACGTGGTTT R: AGCCCTGGTGAAATGTAG		NM_001146142.2	148	61
*SOX10*	F: TCTGAAGACCACCACTGCCTCTC R: CTTGACCTTGCCCATCTCTCCATTC		NM_204792.2	99	60
*CDX2*	F: AAACCAGGACGAAGGACAAATACCG R: GGTGATATAGCGGCTGTAGTGGAAC		NM_204311.2	92	60
*ISL1*	F: TCCGAGGGTCATCAGGGTTTGG R: TGGGTTGCTGCTGCTGAAGTTG		NM_205414.2	92	60
*PAX6*	F: CTATCCCGATGTGTTTGCGAGAGAG R: CTGGGAGTGTTGCTGGCTTGTC		NM_205066.2	150	61
*SOX2*	F: AAACCAAGACCCTGATGAAGA R: ATCCCATAGCCTCCGTTG		NM_205188.3	175	61
*OLIG2*	F: GCTTCAAGTCCTCGTCGTCGTC R: CGGCTCCGTCATCTGCTTCTTG		NM_001031526.1	80	61
*SMAD3*	F: AGACGGCACATCGGAAGAGGAG R: AATGGCACTGTCACTAAGGCACTC		NM_204475.2	81	61
*GATA6*	F: AGAGAGCACCAGTCCCGAAAGC R: ACACCAGTGATCCTGCCTGACG		NM_205420.2	121	61
*SOX9*	F: GCTGTGGAGGCTGCTGAATGAG R: CCTGCGTGGTTGGTACTTGTAGTC		NM_204281.2	112	60
*HNF4A*	F: GCCAACCTCAACACCTCCAACAG R: TCCTCACGCTCCTCCTGAAGAAG		NM_001030855.2	133	61
*MKX*	F: GAACACAGTCAGGCAACCAGACC R: CCATCTTCAGAGCACGAGTCATCAC		XM_015282064.4	120	60
*THRB*	F: AATCAGTGCCAGGAATGTCGCTTC R: GCCTCTTGCTGTCATCCAACACC		XM_015282064.4	82	61
*NFIA*	F: GGAGCCGTTCTATACAAGCCAAGG R: GAAGGCGAGGGACTGCTGAAAC		NM_205273.2	141	60
*ZBTB16*	F: TCCACCGCAACAGTCAGCATTAC R: CGTAGAGCAGGTCATCCAAGTCTTC		NM_001321488.2	126	61
*GAPDH*	F: GCCATCACAGCCACACAGA R: TTTCCCCACAGCCTTAGCA		NM_204305.2	120	60 / 61

### Statistical analysis

Relative expression differences between consecutive stages were calculated, and a t-test was performed in GraphPad Prism 7 (GraphPad Software, San Diego, CA, USA). The differences were considered to be statistically significant at a *P*-value < 0.05.

## Results

### Global view of transcriptome during chicken early development

**Fig. 1:**
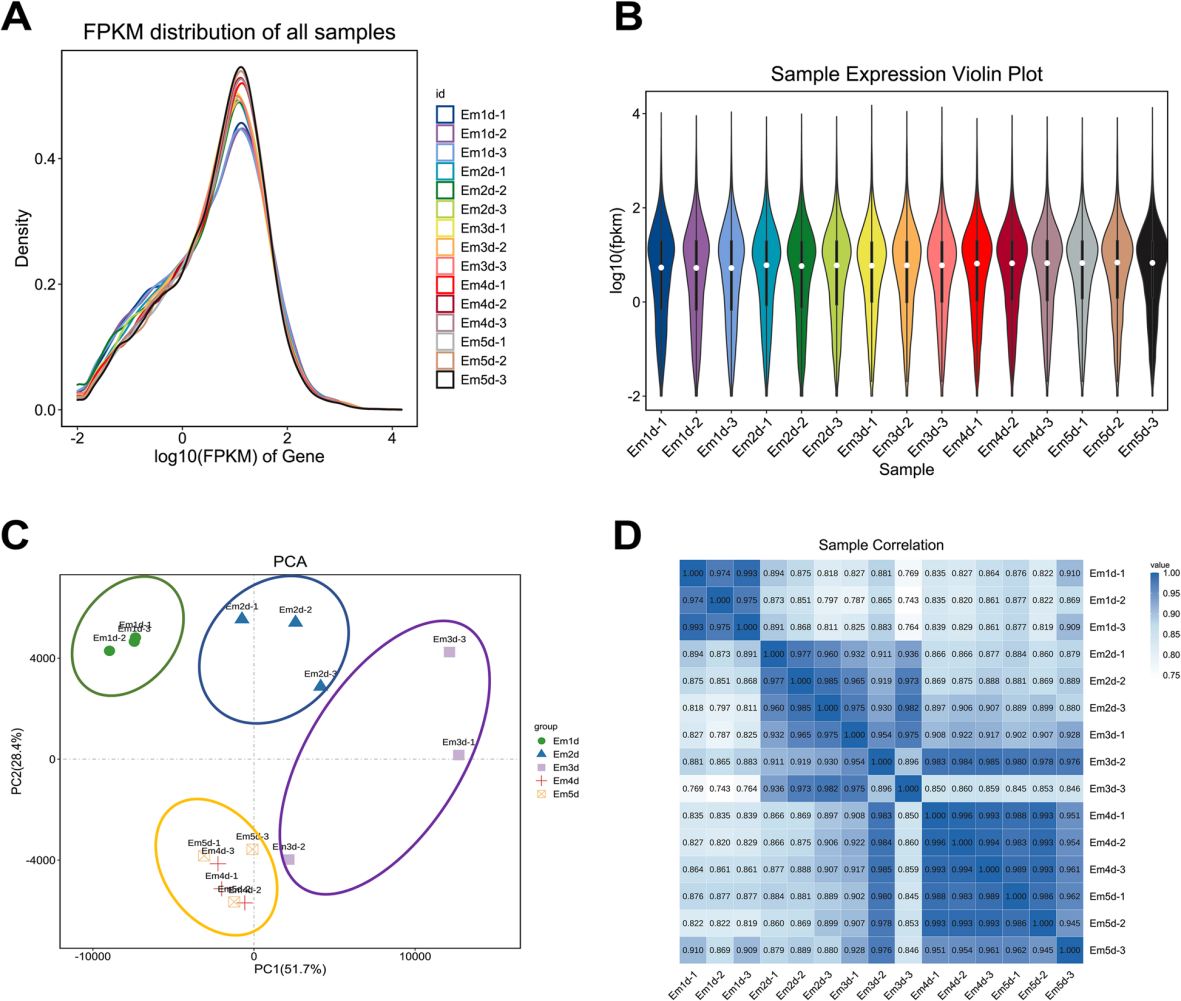
Overview of gene expression during early development in chicken. **A** The density distribution of mRNAs was according to log10 (FPKM); **B** The 15 Samples expression (Em1d-1, Em1d-2, Em1d-3, Em2d-1, Em2d-2, Em2d-3, Em3d-1, Em3d-2, Em3d-3, Em4d-1, Em4d-2, Em4d-3, Em5d-1, Em5d-2, Em5d-3) violin plot, which was replaced by log10 (FPKM). **C** Principal components analysis reveal strong clustering associated with different stages of embryonic development. **D** Sample relationship heatmap plot revealed exact stage of development except for Em3d-2. Dark blue represents strong correlation and light blue represents weak correlation, each column and row correspond to one sample’s relationships with the other 15 samples including itself

To better understand regulation of chicken early development, we performed a comparative transcriptomic analysis. Transcriptome sequencing resulted in a total 813 million raw data for all samples. After removing reads of adapter, reads of poly A and low-quality with a quality score < 20, more than 807 million high-quality reads were remained for further analysis. Reads from each sample were aligned to the chicken reference genome (Supplementary Figure S1). The average number of raw data, filter data, GC content, number of mapped reads and mapping rate for samples are shown in Supplementary Table S1. From each stage, a total number of 93.90–95.21% reads were successfully mapped. Approximately 80% of transcripts exhibited great gene coverage (Supplementary file Figure S2). The number of genes displayed saturation tendencies, and all samples were distributed in a homogeneous and random manner. (Supplementary Figure S3). Original gene read counts were normalized using the FPKM (Fragments per kilo-base of exon per million fragments mapped) method. Figure 1A represents the FPKM distribution of mRNAs, while Fig. 1B depicts the expression of different samples as a violin chart (Fig. 1B). Principal components analysis is useful for exploring the distance relationship between samples. The 15 samples were divided into four parts, which showed satisfactory repeatability and strong clustering associated with development stage, excluding sample Em3d-2 (Fig. 1C). To be clear, although the principal component analysis shows that sample Em3-2 is more similar to day4 and day5, the correlation analysis presents a greater convincing result that Em3d-1, Em3d-2 and Em3d-3 are good replicates with > 0.85 Pearson correlation coefficient. Additionally, with the low degree of outlier that would not affect the following analysis, we did not eliminate the sample Em3d-2. Then, we established a relationship cluster heatmap plot to reflect the relationship between samples intuitively (Fig. 1D). Data showed a reliable clustering effect, which ensured the veracity of the subsequent analysis except for the sample Em3d-2.

### Identification of DEGs during early development of chicken

**Fig. 2:**
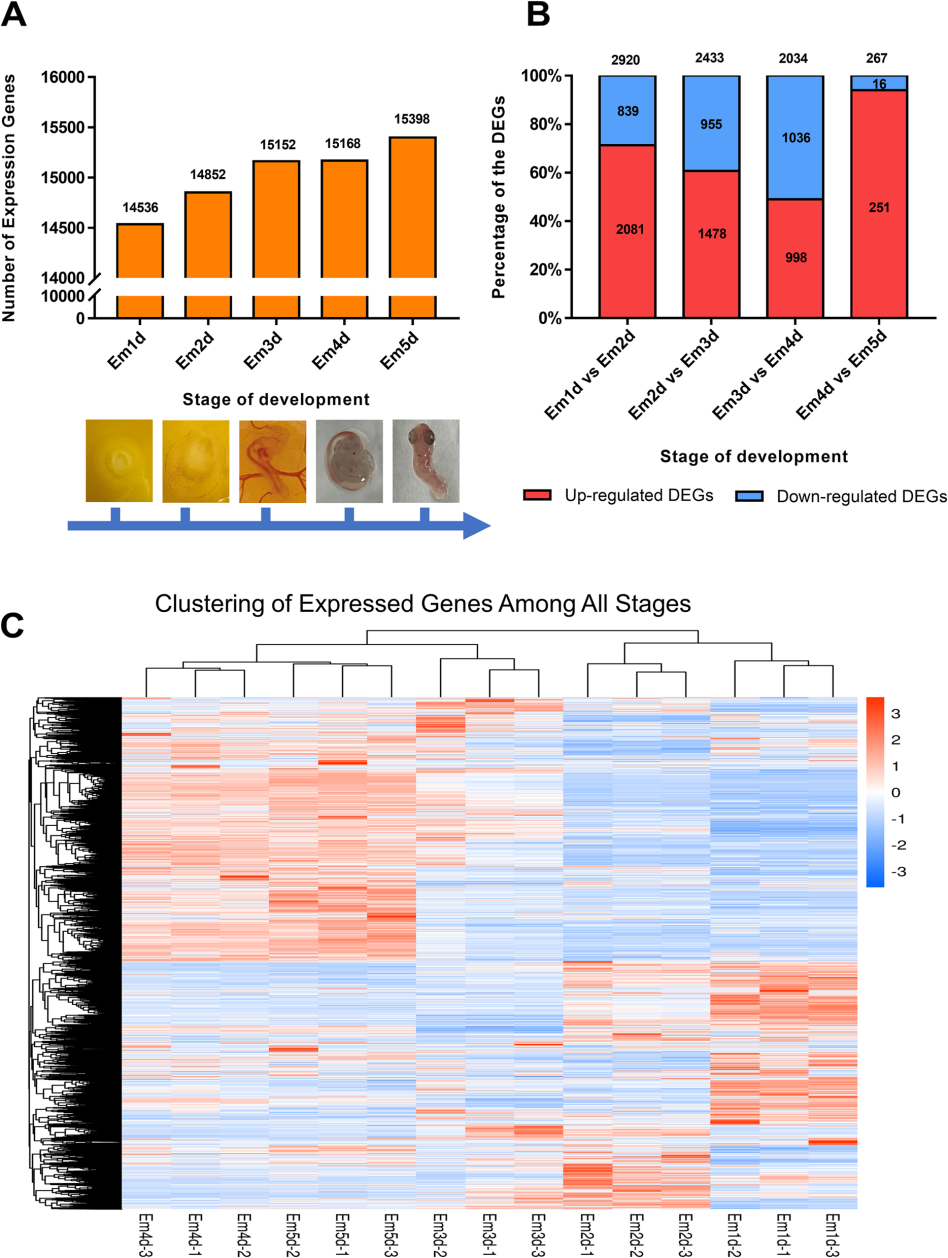
The differential expression analysis of genes. **A** Mean number of expressed genes of three replicates identified at each development stage. **B** The number of differentially expressed genes (DEGs) for comparison of each stage with the next stage. **C** Cluster analysis of gene expression. Embryos at different stages were clustered into two distinct clusters. One cluster contains replicates of Em1d and Em2d, two-stages. While Em3d, Em4d, and Em5d into the second cluster. This indicates a major shift in the gene expression from 3 stage onward

To investigate embryonic development alterations in the gene expression pattern during the early stages, differential gene expression analysis was conducted among the five developmental stages in chicken using the software package DESeq2. Generally, the expression of 18,325 distinct genes was identified, including 847 novel genes. The highest number of expressed genes (15,398) occurred on day 5 of embryo, while Em1d sample contained the lowest number of expressed genes (14,536) (Fig. 2A). Subsequently, DEGs (*FDR* < 0.05 and Fold Changes > 2) were identified by comparing two consecutive developmental stages. The number of DEGs varied from 267 (251 upregulated and 16 downregulated) between 5 and 4-day of embryo, to 2920 (2081 upregulated and 839 downregulated) between 2 and 1 day of embryo (Fig. 2B). Interestingly, up-regulation dominated the genes expression patterns in all comparisons, except for the transition from Em3d to Em4d stages, while 51% of genes showed down-regulation. Additionally, Hierarchical clustering of DEGs, based on log 2-transformed expression values, was able to cluster these stages into distinct groups (Fig. 2C). Unexpectedly, stages Em1d and Em2d were clustered together in one group, while Em3d, Em4d and Em5d were grouped in a separate cluster, indicating that a major shift occurred in that situation.

### Transcription factor expression patterns during early development of chicken embryos

**Fig. 3:**
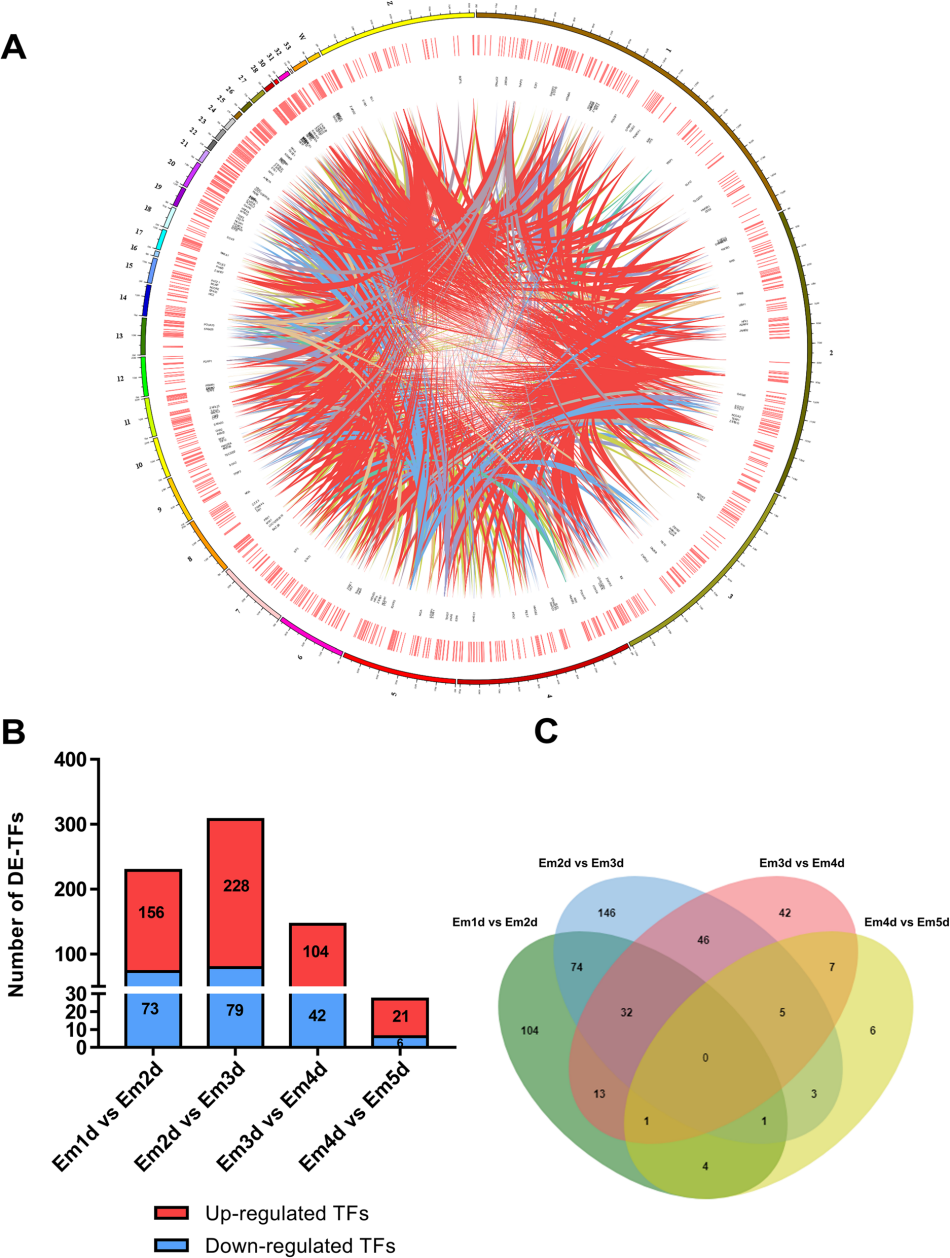
Analysis and detection of DE-TFs during early chicken embryogenesis. **A** CIRCOS visualization of TFs at the genome-wide level; from outside to inside: Karyotype of the chicken genome, expression of TFs, specific TFs symbol and linkage of TF family. **B** DE-TFs were identified during the transition of the embryo from Em1d to Em5d. The red color indicates the up-regulated TFs, while the blue color indicates the down-regulated TFs. **C** The status of common TFs involved in each transition. DE‐TF: differentially expressed TF; TF: transcription factor

To visualize the landscape of transcription factors at the genome-wide level, we have constructed a CIRCOS diagram (Fig. 3A). From the results, a total of 1134 TFs (Supplementary Table S2) were distributed in 32 normal chromosomes and 2 sex chromosomes (Z and W), where 41 TFs were located in Z chromosome but only 6 in W chromosome. The fact that TFs were abundant in the left hemisphere suggests that their location in the genome was not random. Then, to explore the different contributions of TFs in different stages of early embryonic development, we identified multiple TFs in variation of expression (Fig. 3B). The most different expression TFs (DE-TFs) change was observed in the transition from Em2d to Em3d, while fewer and fewer counts of DE-TFs are getting involved in later stages, where expression of only 27 TFs changes during the transition from Em4d to Em5d. Furthermore, to investigate TFs that express commonly between successive stages in embryo development throughout the early embryonic period, we performed Venn on DE-TFs at different stages. Figure 3C shows that 32 DE-TFs are expressed from Em1d to Em4d, while 5 TFs from Em2d to Em5d. More importantly, transcription factor OSR2 and EOMES were observed that significantly different expressing among all stages, from Em1d to Em5d. Additionally, 164 TFs showed constant and highly expressed through all stages (Supplementary Table S3).

**Fig. 4:**
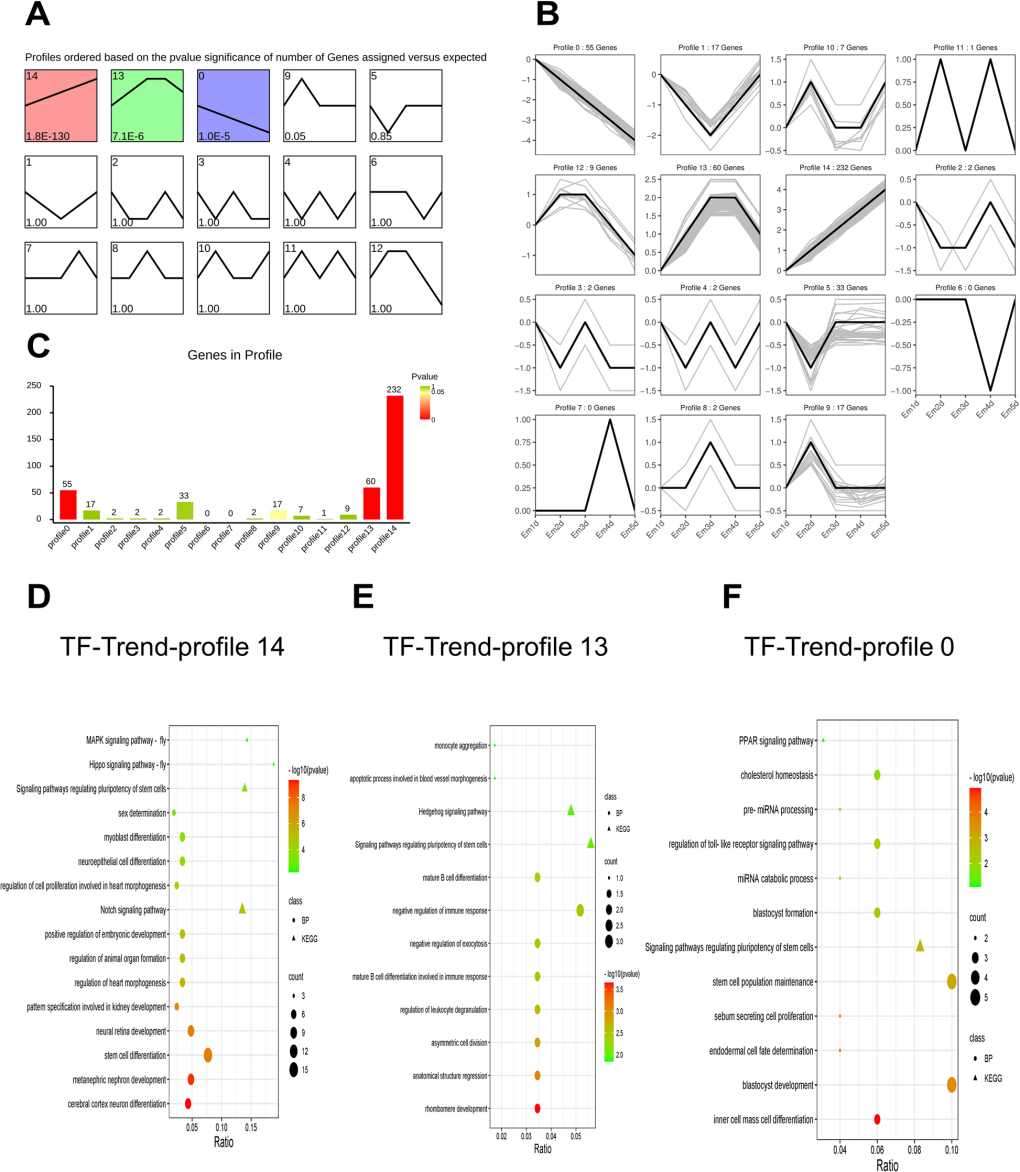
The sample time series analysis of DE-TFs. **A** Distribution trend of differential transcription factors, color means significant difference (*P* < 0.05), gray means not significant (*P* > 0.05); **B** The time series line of differential transcription factors. **C** Representative terms of GO and KEGG using profile-14 TFs. **D** Representative terms of GO and KEGG using profile-13 TFs. **E** Representative terms of GO and KEGG using profile-0 TFs. KEGG pathway database was used to analyze the data

Then, we profiled the time series analysis to illustrate the dynamic changes of TFs. All TFs were clustered into 15 trends, of which three trends appeared significant (*P* < 0.05) (Fig. 4A). The time-series line of differential gene expression is shown in Fig. 4B. The overall TF expression trend was classified as either rising or falling. Generally, a total of 232 DE-TFs were significantly enriched in always up-regulation trend (profile 14) and 55 DE-TFs were consistently down regulation (profile 0) (Fig. 4C). These findings demonstrate the gene expression status of embryo development in the early stages.

### Function enrichment analysis of differential expressed transcription factors

**Fig. 5:**
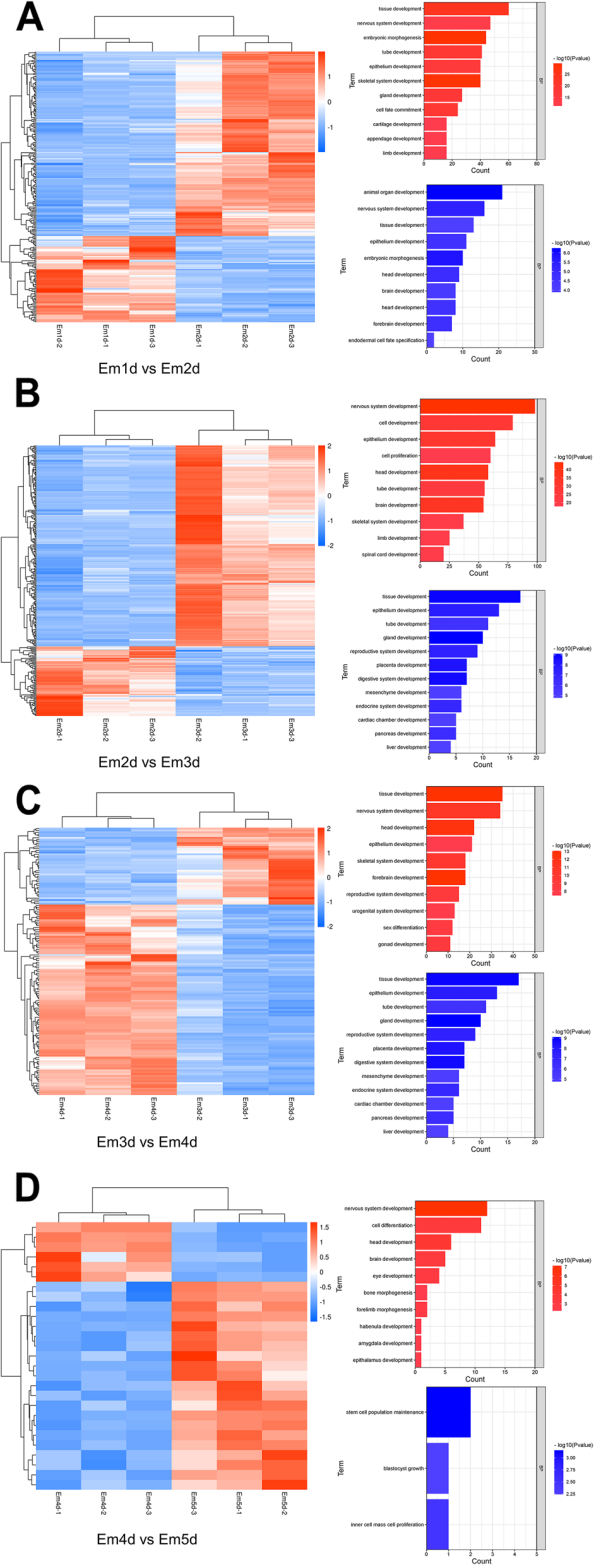
Clustering based on DE-TFs and the annotation of the DE‐TFs. Distinct clusters in all stages are evident based on the expression of DE‐TFs. GO of TFs was also provided alongside each cluster. **A** Transition from Em1d to Em2d, **B** transition from Em2d to Em3d, **C** transition from Em3d to Em4d, **D** transition from Em4d to Em5d. DE‐TF: differentially expressed TF; GO: gene ontology; TF: transcription factor

Cluster analysis and GO enrichment analysis were used to explore the differential expressed TFs. As shown in Fig. 5, two distinct clusters were observed when the later stage compared with the previous stage, indicating significant differences in the regulation of transcription factor expression. Therefore, we performed GO enrichment analysis between the biological processes for up-regulated and down-regulated TFs groups separately. As we known, the transcription processes, biosynthetic processes, and binding processes are the main processes controlled by these TFs, thus we have excluded these annotations from the list of biological processes. The top 10 enrichment significant terms (*P* < 0.05) in the Biological Process section were displayed in Fig. 5. Obviously, early stages of embryonic development have a large number of biological processes, while a limited terms are identified at later stages. For instance, at the transition from Em1d to Em2d stages (Fig. 5A), about 30% of up regulated TFs are related to skeletal system development, epithelium development, nervous system development and embryonic morphogenesis (Supplementary Table S4), especially containing cell fate commitment related TFs (*NKX2-5*, *PRRX1*, *LEUTX*, *SOX9*, *SOX8*, *NR2F2*, *SATB2*, *TBX5*, *HOXD10*, *PROX1*, *PAX6*, *ZNF521*, *NR221*, *GCM1*, *PITX1*, *AR*, *FOXA1*, *GLI3* ), while 20% of down regulated TFs are related to embryonic morphogenesis and epithelium development including *LHX1*, *GBX2*, *MSX1*, *EOMES*, *OTX1*, *ZIC3*, *SOX17*, *SALL4*, *SP9*, *SCX* (Supplementary Table S4). In the comparison between Em2d and Em3d stages (Fig. 5B), we found that the counts of up regulated TFs in limb development, tube development, brain development, head development and nervous system development are enormous growth (Supplementary Table S4). In contrast, down regulated TFs were involved in reproductive system development and placenta development (*GATA2*, *HNF1A*, *OVOL2*, *PRDM1*, *GCM1*, *GATA4*, *ARID5B*, *FOXA1*, *VDR*, *TBX3*). Gonad development and sex differentiation such as *LHX9*, *SOX9*, *SOX8*, *OSR1*, *HOXA10*, *FOXL2*, *AR*, *NHLH2*, *HOXA11*, *ZFPM2* were up regulated during Em3d to Em4d stages, while TFs (*HNF1A*, *FOXA2*, *HAND1*, *PITX2*) controlling mesenchyme development were observed that down regulated. Moreover, fewer significant different expressed TFs were detected at the stages from Em4d to Em5d, *MYOD1*, *THRB*, *NR4A2*, *RORB*, *EOMES*, *TBR1*, *SOX14*, *OSR2*, *RUNX2*, *NFATC1*, *HELT* involved in cell differentiation were up-regulated, while down regulated *LIN28A* and *SALL4* were enriched in stem cell population maintenance (Supplementary Table S4).

Additionally, KEGG enrichment analysis and GO enrichment analysis were performed to investigate the TFs with the same expression pattern in a time line (Fig. 4D-F). From the KEGG and GO results, we found that the trend profile 14 was enriched in regulating pluripotency of stem cell and cell differentiation including myoblast differentiation, stem cell differentiation and neuron differentiation (Fig. 4D). Especially, MAPK signaling pathway and Hippo signaling pathway both involved in differentiation and stemness. However, in the trend profile 0, TFs were mainly enriched in stem cell population maintenance and blastocyst development (Fig. 4F).

### Dominant transcription factor families in early embryo development

**Fig. 6:**
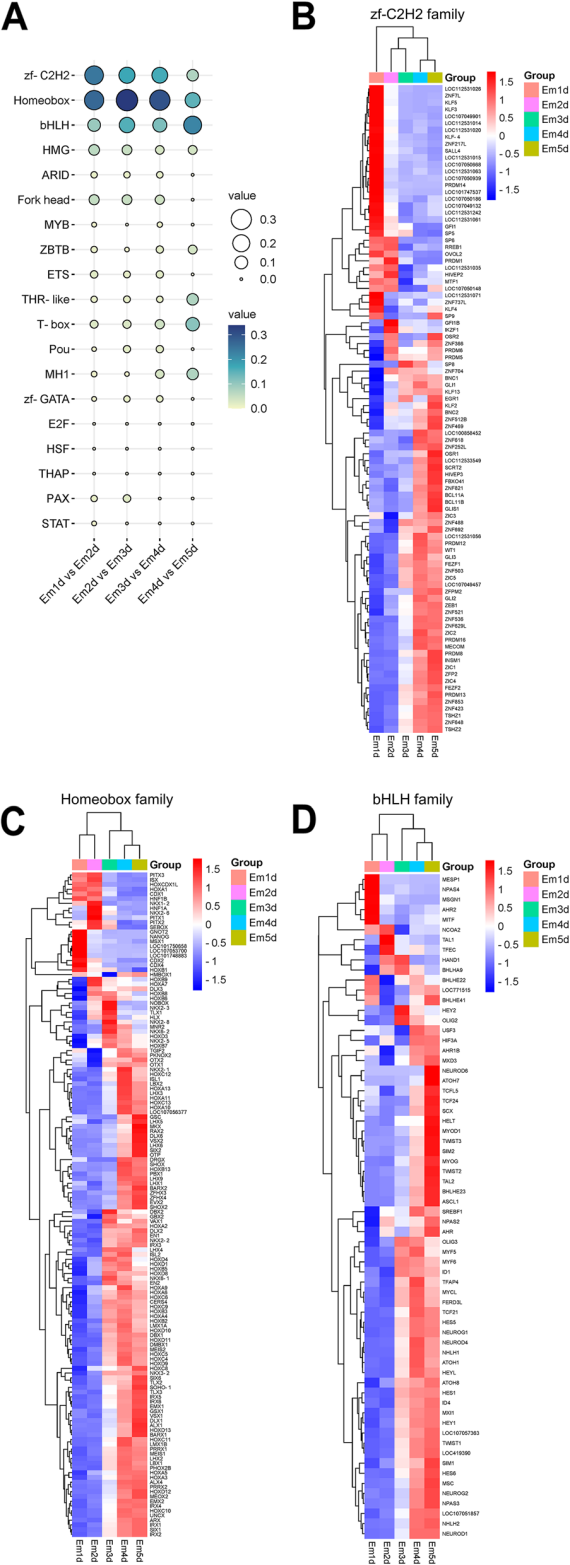
Dominant TF families in early chicken embryogenesis based on DE-TFs in successive developmental stages. Zf-C2H2, Homeobox, and bHLH families are the top 3 TF families. **A** The rate of the top 20 TF families in each transitional stage of embryo. **B** The dynamic expression of the zf-C2H2 family. **C** The dynamic expression of the Homeobox family. **D** The dynamic expression of the bHLH family

Based on structure of DNA-binding domains that are important evolutionary units mediating the specificity of the TF-DNA interaction, transcription factors can be grouped into different families [32]. According to our data, we analyzed the distribution of TF families of DE-TFs at five stages in embryos and found that there were different distributions in the top three largest TF families. The bubble plot (Fig. 6A) showed that zf-C2H2, Homeobox and bHLH are three dominant TF families (Supplementary Table S5). Interestingly, zf-C2H2, as best known and largest TF family in human [33], is also represent the major class of chicken transcription factors. On the other hand, however, we found that Homeobox family occupied the largest portion and was expressed during the whole stages, while bHLH family contain fewer TFs expressing mostly occurred in Em4d and Em5d (Fig. 6B-D).

### Network construction and analysis of hub transcription factors at each stage of embryonic development

**Fig. 7:**
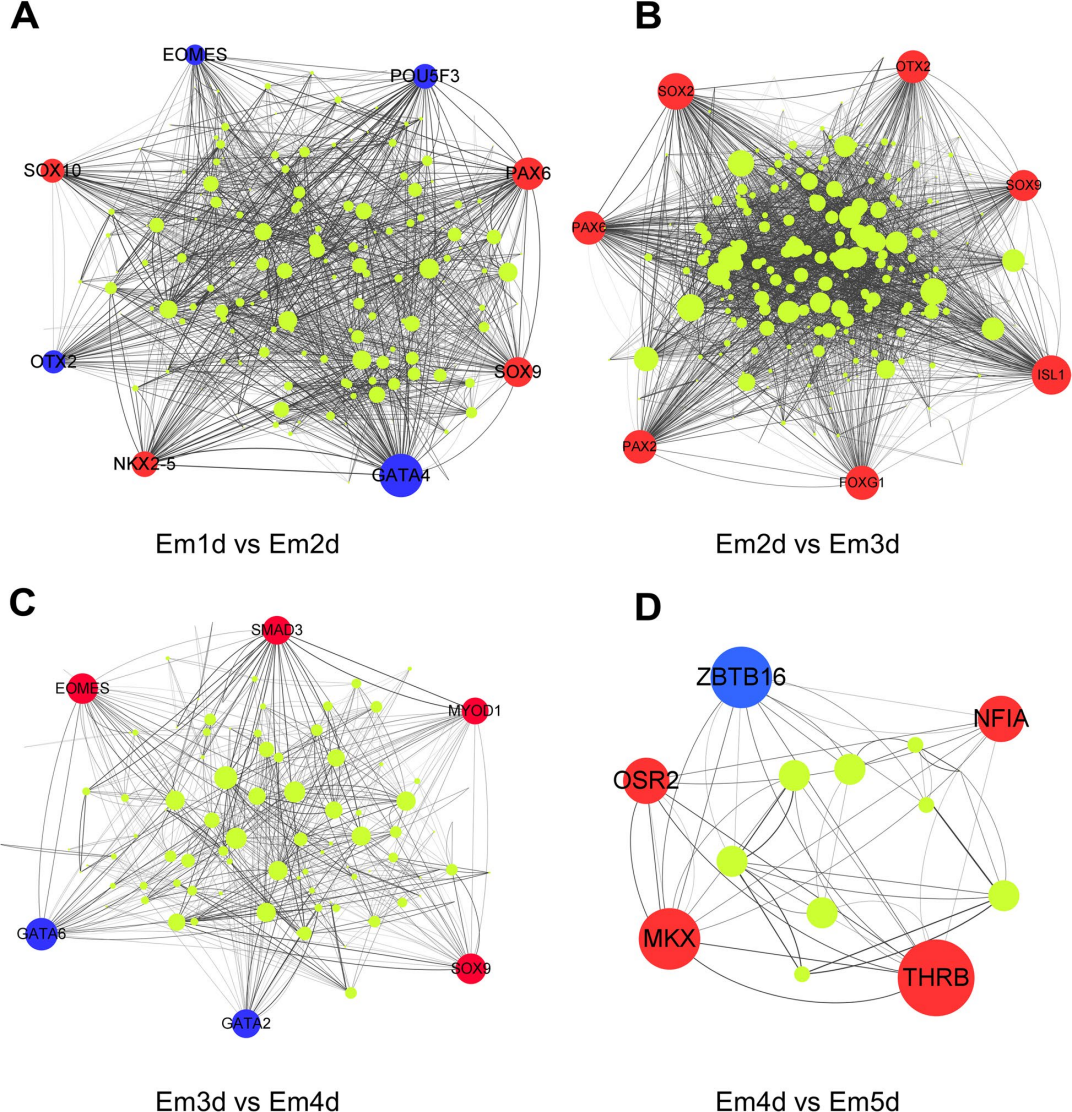
Protein–protein networks of regulatory TFs at early stages of embryonic development. The networks were constructed for the transition from **A** Em1d to Em2d, **B** Em2d to Em3d, **C** Em3d to Em4d, **D** Em4d to Em5d. Red and blue color indicate up- and down-regulation, respectively. The width of edge was calculated by combined-score

**Fig. 8:**
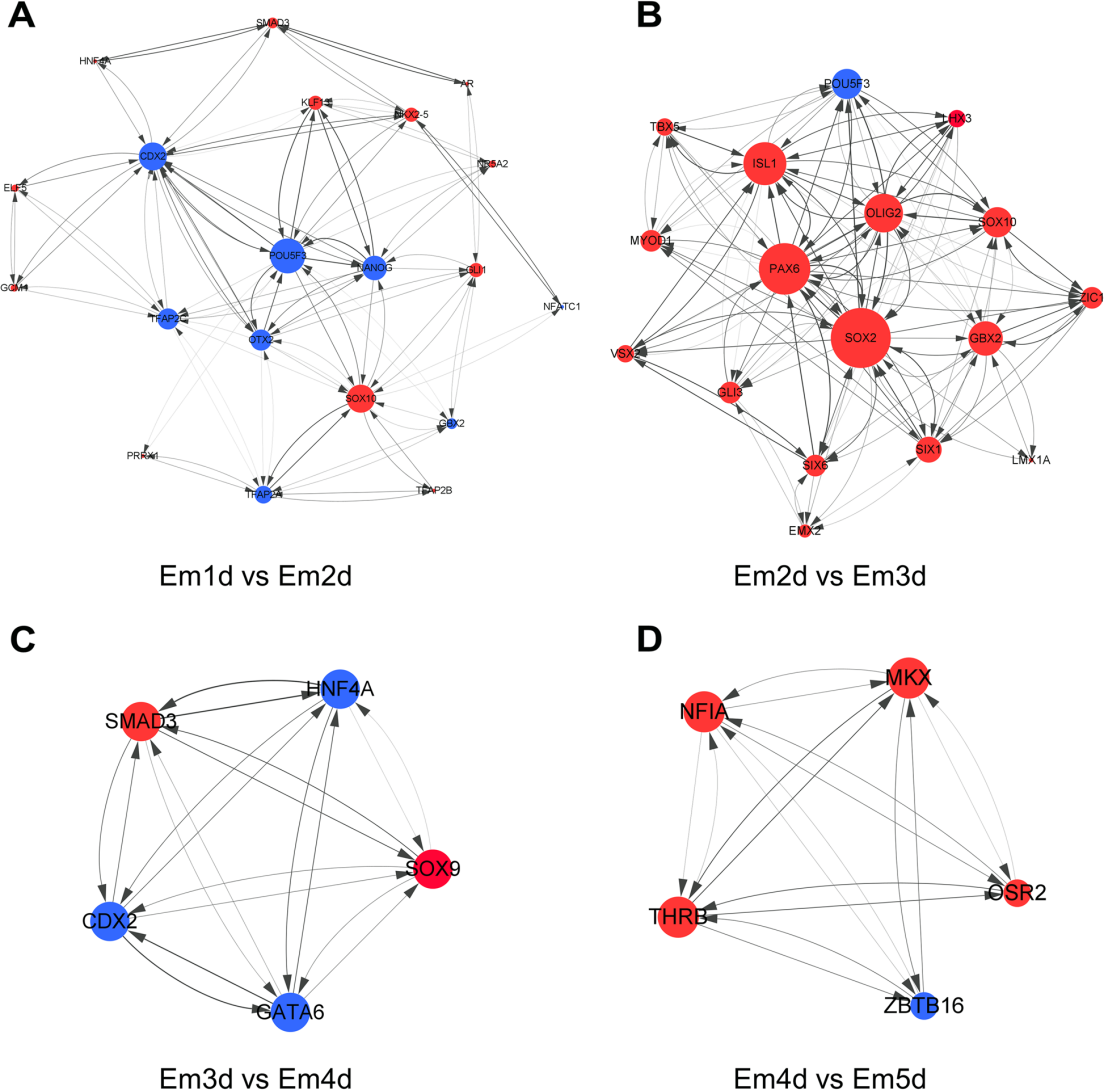
The core regulatory network information involved in different stages transition during chicken embryogenesis. Directed and autoregulation by different TFs at **A** Em1d to Em2d, **B** Em2d to Em3d, **C** Em3d to Em4d, **D** Em4d to Em5d are given in the networks. Red nodes are up-regulated while blue nodes are down-regulated in the network constructed for any given stages

To further identify the function of the co-expressed TFs in different stages and investigate the hub TFs, we have constructed co-expression network. Additionally, a core regulatory networks (Fig. 7) were extracted from the whole network analysis through MCODE algorithm. During the transition from Em1d to Em2d stage, we have detected *EOMES*, *POU5F3*, *PAX6, SOX9*, *GATA4*, *NKX2-5*, *OTX2* and *SOX10* as key factors for regulation of TFs (Fig. 7A). The network analysis showed that *GATA4* has the highest number of interactions with other TFs and highly expressed in Em1d stage. Importantly, *POU5F3*, *NANOG* and *CDX2* were also detected as hub genes in the core network (Fig. 8A).

As shown in Fig. 7B, *SOX2*, *OTX2*, *SOX9*, *ISL1*, *FOXG1*, *PAX2* and *PAX6* play a key role during transition from Em2d to Em3d stage, which all were up-regulated. However, the core regulatory network analysis at these stages indicated that *ISL1*, *PAX6*, *SOX2* and *OLIG2* are the hub proteins (Fig. 8B).

When it comes to transition of Em3d to Em4d, the embryos are mostly regulated by *SMAD3, MOYD1, SOX9, GATA2, GATA6* and *EOMES* with the highest number of connections (Fig. 7C). On the other hand, the core regulatory network detected not only *SMAD3, SOX9* and *GATA6* as hub genes, but also *HNF4A* and *CDX2* (Fig. 8C). Moreover, the pattern of expression during this transition is that *SMAD3* and *SOX9* are up-regulated while *GATA6*, *HNF4A* and *CDX2* are down-regulated.

In the last period, the least differential expressed TFs resulted in that *NIFA*, *THRB*, *MKX*, *OSR2* and *ZBTB16* are detected as hub genes for both PPI network and core regulatory network (Figs. 7D and 8D). Besides, only *ZBTB16* was down-regulated.

**Fig. 9:**
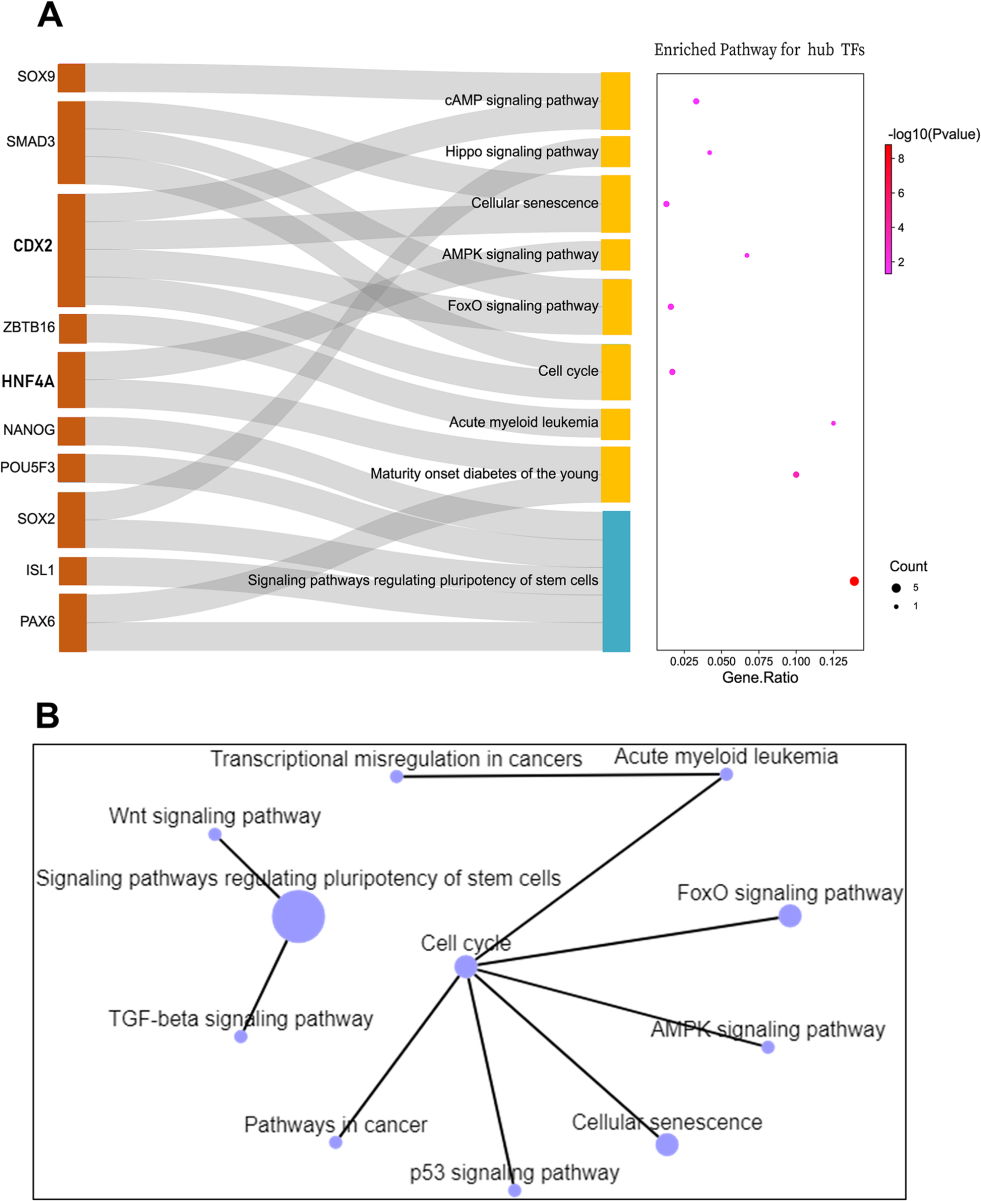
The KEGG pathway analysis of hub TFs during chicken embryogenesis using the KEGG pathway database. **A** The Sankey plot showing the enriched pathway for hub TFs. **B** Network analysis of enriched pathway of hub TFs

Nevertheless, the top significantly enriched pathways particular to the hub TFs include the signaling pathways regulating pluripotency of stem cells, cell cycle, FOXO signaling pathway, AMPK signaling pathway, Hippo signaling pathway and cAMP signaling pathway ect. Also, the network of key pathways was constructed and was displayed in Fig. 9B. We identified two clusters with the predominant clusters belonging to regulation of pluripotency of stem cells and cell cycle signaling pathways as depicted in Fig. 9A. From Fig. 9A, it is shown that TFs such as *NANOG*, *POU5F3*, *SOX2*, *ISL1*, and *PAX6* were the one which are involved in regulation of pluripotency of stem cells, whereas TFs such as SOX9, SMAD3, CDX2, ZBTB16, and HNF4A were the one associated with cell cycle signaling pathways.

### Validation of the hub TFs in embryonic development by RT-qPCR

**Fig. 10:**
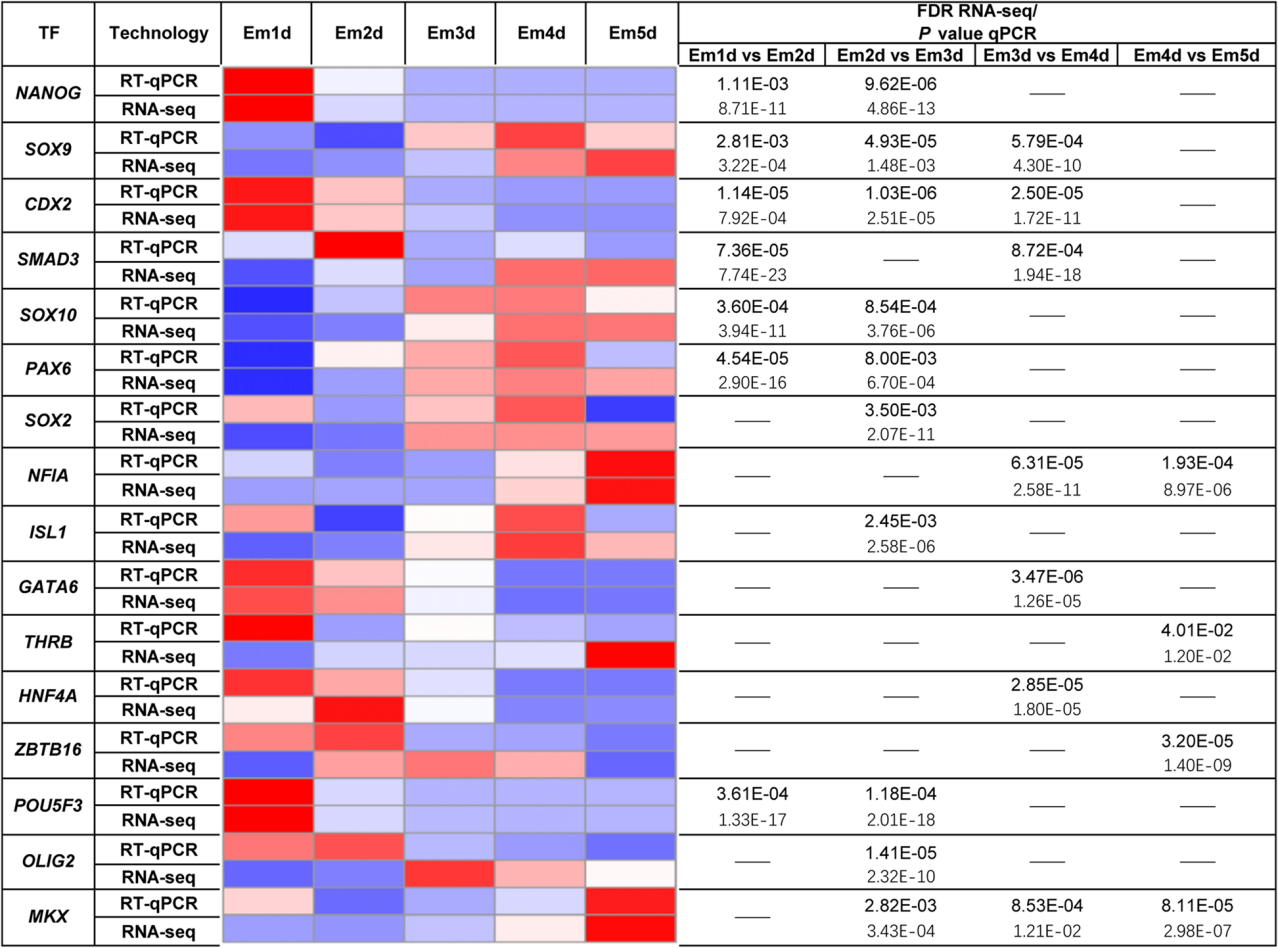
The validation of selected hub TFs by RT-qPCR: Heatmap are constructed of qPCR and RNA-seq data for 16 selected genes. The relationship between the relative expression levels of RNA-seq and qPCR data (mean-centered log2 expression values) are shown as a heatmap. The color red denotes higher gene expression levels, whereas the color blue denotes lower gene expression levels when compared to the mean of all samples., respectively (from 2 over 0 to − 2). Statistical differences are represented by FDR for the RNA-seq data and by *P* value for qPCR data (*P* < 0.05)

To validate the 16 selected hub TFs at different stages during early embryonic development, RT-qPCR was conducted to illustrated the gene expression shown in Fig. 10. Differences in embryonic TF expression at each stage profiled by RNA-seq results were confirmed for all of 16 genes by qPCR (*P* value < 0.05). Evidently, comparable patterns and similar trends in gene expression could be observed for the key TFs. These findings could validate the specific role of these TFs.

## Discussion

Chicken have long been regarded as an ideal model for virology, physiological and behavioral traits, immunology, biotechnology and developmental biology [34–38]. In light of the importance of the chicken to human societies around the world, genetic diversity and gene regulatory of the chicken (Gallus gallus) is of great interest [39]. Since that vast majority of biological processes, from development to homeostasis maintenance, from cell cycle to cell differentiation, are tuned by differential gene expression [40], understanding expression patterns of TFs is fundamental important in early embryo development. Of note, studies about TF regulation in embryo cover many domestic animals including, porcine, equine, bovine and sheep [41–44]. However, the whole transcription factors landscape of early chicken embryo remains unclear. Here, in our study, we categorized expressed TFs based on RNA-seq data regarding chicken embryos from Em1d to Em5d.

The embryonic gastrulation and then organogenesis all take place in vitro after oviposition. Somitogenesis progress is noticeable during the first 1–5 days of incubation [45, 46], therefore, E1–E5 is a crucial era in developmental biology research. Comparative analysis of gene expression pattern among successive stages showed that up-regulation of gene is indeed the main molecular events. Also, we have found that gene expression pattern is dramatically altered during the transition from Em2d to Em3d.

To date, a total of 1134 TFs were discovered in chicken. Notably, in this current study, we identified 1097 TFs during early embryonic development, which are not randomly distributed in genome but should topologically organized. Previous studies [47, 48] have suggested that genes with particular expression pattern are sometimes found in contiguous regions of the genome (named gene-expression neighborhoods), and the phenomena that remote regulatory elements control genes activity or expression other than the one they overlap with or are nearest to is extremely common genome-wide. In addition, the result of this study demonstrated that Zf-C2H2, Homeobox, and bHLH are three dominantly expressed TF families in early embryo development. Forming the largest TF family in animal kingdom, Zf-C2H2 is the most widespread element of various DNA-binding domains and contribute most of the diversity to the motif collection, which regulating development and differentiation in the early embryonic stage [1, 49–51]. The Homeobox family contains homeodomain of about 60 amino acids coded by *Hox* genes, which are essential transcription factors for all aspect of development owing to their major roles in the determination of cell fates and cell differentiation [52]. The hub TFs such as *NANOG*, *CDX2*, *ISL1*, and *MKX* in chicken embryo development are belong to Homeobox family (Table 2). Accumulating evidences show that the bHLH factors correlate with multipotent and proliferative state and regulate fate determination of somatic cells into neurons [53–55]. More importantly, the cranio-caudal polarity, as well as that of specific cell groups within the somites, is determined by transcription factors of the bHLH and homeodomain type. According to our study, it is found that the bHLH factors were highly expressed in Em4d and Em5d, which have more responsibility for nervous system development. Additionally, 164 constant and highly expressed TFs were observed in all stages, indicating that these TFs are common and necessary in development (Supplementary Table S3).

**Table 2:** List of detected hub TFs in chicken embryo development

Symbol	NCBI Gene ID	Chromosome	TF family
POU5F3	427,781	17	Pou
NANOG	100,272,166	1	Homeobox
SOX10	395,573	1	HMG
CDX2	374,205	1	Homeobox
ISL1	396,383	Z	Homeobox
PAX6	395,943	5	PAX
SOX2	396,105	9	HMG
OLIG2	428,612	1	bHLH
SMAD3	395,132	10	MH1
GATA6	396,390	2	zf-GATA
SOX9	374,148	18	HMG
HNF4A	419,198	20	RXR-like
MKX	771,284	2	Homeobox
THRB	396,431	2	THR-like
NFIA	396,210	8	NFI
ZBTB16	419,759	24	Zf-C2H2

Embryonic development related TFs have different regulatory effects at different stages of development. Simultaneously, there are significantly change in TF expression at different developmental times. Therefore, time series analysis was utilized to characterize TF expression and disclose the law of embryonic development at various stages. Subsequently, differentially expressed TFs are clustered into three mainly trend profiles. Different TFs in the same trend were analyzed for their involvement in the same biological process using functional enrichment analysis. Multiple development-related terms were considerably enriched when the GO and KEGG analysis was applied to the increasing trend, such as MAPK signaling pathway, Hippo signaling pathway, PPAR signaling pathway and pathways regulating pluripotency of stem cells. Notably, it was discovered that active p38-MAPK signaling is required for blastocyst development [56]. Interestingly, not only the involvement of the FGF/MAPK signaling pathway in early neural crest induction during gastrulation has been elucidated, and it also plays many roles in the formation of ectodermal tissues [57]. The HIPPO signaling pathway is highly conserved across animal species ranging from drosophila to mouse [58]. Additionally, Hippo signaling is important in early embryonic development and positively or negatively regulates development of multiple tissues/ organs [59]. Besides, increasing evidences highlight the functional importance of PPAR related gene expression during embryonic development and the maintenance of embryonic stem cells’ pluripotent state [43, 60]. Notwithstanding, the mechanisms by which signaling pathways influences development of embryo are not entirely clear, and further studies are needed to supplement the gap.

Gene regulation networks (GRNs) control a variety of developmental and cellular functions including cell differentiation and cell fates by regulating gene expression [61]. Transcription factors control the expression of regulatory genes and all other genes by means of regulatory interactions [62, 63]. Therefore, it is important to explore hub TFs in the early embryo development by constructing gene regulation networks. In our networks, we found the main regulators of transition in the early stages from Em1d to Em5d. In our results, *NANOG*, *POU5F3*, and *CDX2* were found that play a pivotal role in the core regulatory network of transition from Em1d to Em2d. Especially, *NANOG* and *POUV* are involved not only in fundamental events such as zygotic genome activation (ZGA), but also in the acquisition of pluripotency that occurs at stage EGK.VI to EGK.VIII [16]. However, in our presented data, *NANOG* and *POUV* were significantly down regulated thereafter. *CDX2* plays a well-defined role in determining the first lineage decisions and in assigning positional identity during orchestrated process of embryogenesis [64, 65], and is also involved in gut epithelial differentiation and intestinal differentiation [66, 67]. While in the core regulatory network at the stages from Em2d to Em3d, *SOX2*, *OLIG2*, *PAX6*, *ISL1* and *SOX10* are shown interaction with each other, and both participate in central neuronal system development including development of neural crest cell that is important in embryogenesis [68, 69]. In addition, *PAX6* and *ISL1* are required for other neuronal development such as dendrite morphogenesis and pancreatic development [70, 71]. *ISL1* is also known as a marker for cardiac differentiation [72, 73]. Meanwhile, previous studies have shown that *PAX6* is involved in the regulation and development of the eye [74–76]. Network analysis have introduced *SMAD3*, *GATA6* and *SOX9* as hub TFs during the stage from Em3d to Em4d, which are critical players in reproductive development and function [77]. *SMAD3* and *SOX9* was shown highly expression in E4 when PGCs migrate into primitive gonad (develop on ventromedial surface of the embryonic kidney), which promote differentiation of gonad [78–80]. Immunity system and brain development are the main issues in the transition of Em4d to Em5d. For instance, ZBTB16 regulates innate and innate-like lymphoid lineage development [81, 82]. THRB and NFIA are both involved in retina and brain development [83, 84]. However, a few studies have investigated the roles of *ZBTB16*, *THRB*, *NIFA* or *MKX* in chicken. Their functions need to be uncovered through further researches.

## Conclusion

This study first analyzed TFs expression pattern from embryonic development stage Em1d to Em5d through RNA-seq, clustering, enrichment and network analysis. Our comprehensive, unbiased analysis of dynamic TFs change during early embryo development in chicken reveals critical regulatory factors and provide new insights into embryogenesis. Collectively, these results offer a basis resource for further studies.

## Supplementary Information

**Additional file 1: Figure S1. **Overview of RNA-seq mapping in chicken genome. **Figure S2.** Gene coverage of different samples. **Figure S3.** Sample randomness distribution.

**Additional file 2: Table S1.** Data quality assessment in sequencing. 

**Additional file 3: Table S2.** Distribution of TFs in genome. 

**Additional file 4: Table S3.** Common TFs highly expressed in all stages. 

**Additional file 5: Table S4.** Go enrichment of DE-TF in different stages. 

**Additional file 6: Table S5.** Differentially expressed transcription factors (DE-TFs). 

## Data Availability

RNA–Seq raw data in this study are available at NCBI’s Sequence Read Archive (SRA) under the BioProject accession PRJNA850787. Additionally, all data generated or analyzed during this study are included in this published article and its supplementary information files.

## Abbreviations

DEGs: Differentially expressed genes
TF: transcription factor
EGA: embryonic genome activation
ZGA: zygotic genome activation
Inner cell mass: ICM
FPKM: Fragments per kilo-base of exon per million fragments mapped
PAC: Principal component analysis
FDR: False discovery rate
GO: Gene ontology
KEGG: Kyoto encyclopedia of genes and genomes
qRT-PCR: Quantitative real-time polymerase chain reaction
GRNs: Gene regulation networks
