# Supplementary material for: Transcriptomic analysis reveals the dynamic changes of transcription factors during early development of chicken embryo
Source: BMC Genomics. 2022 Dec 13;23:825. doi: 10.1186/s12864-022-09054-x (PMC9746114; doi:10.1186/s12864-022-09054-x)
Supplement: Supplementary file 1 — Additional file 1: Figure S1. Overview of RNA-seq mapping in chicken genome. Figure S2. Gene coverage of different samples. Figure S3. Sample randomness distribution. [file 12864_2022_9054_MOESM1_ESM.pdf]

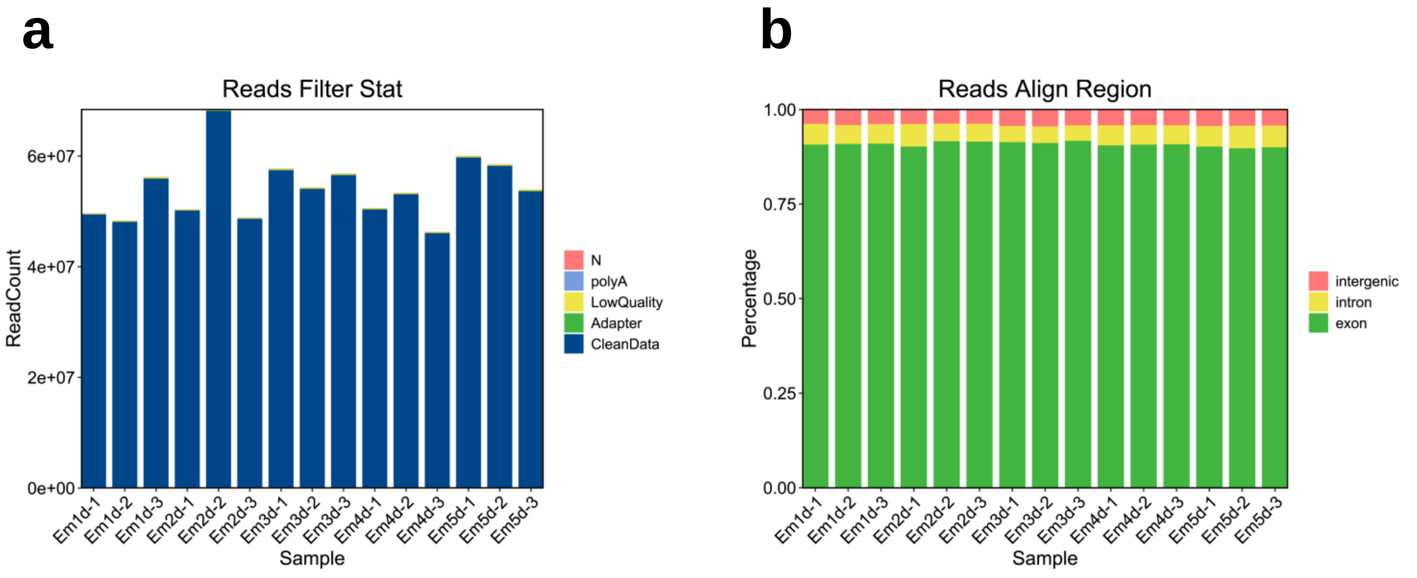

Figure S1. Overview of RNA-seq mapping in chicken (*Gallus gallus*).

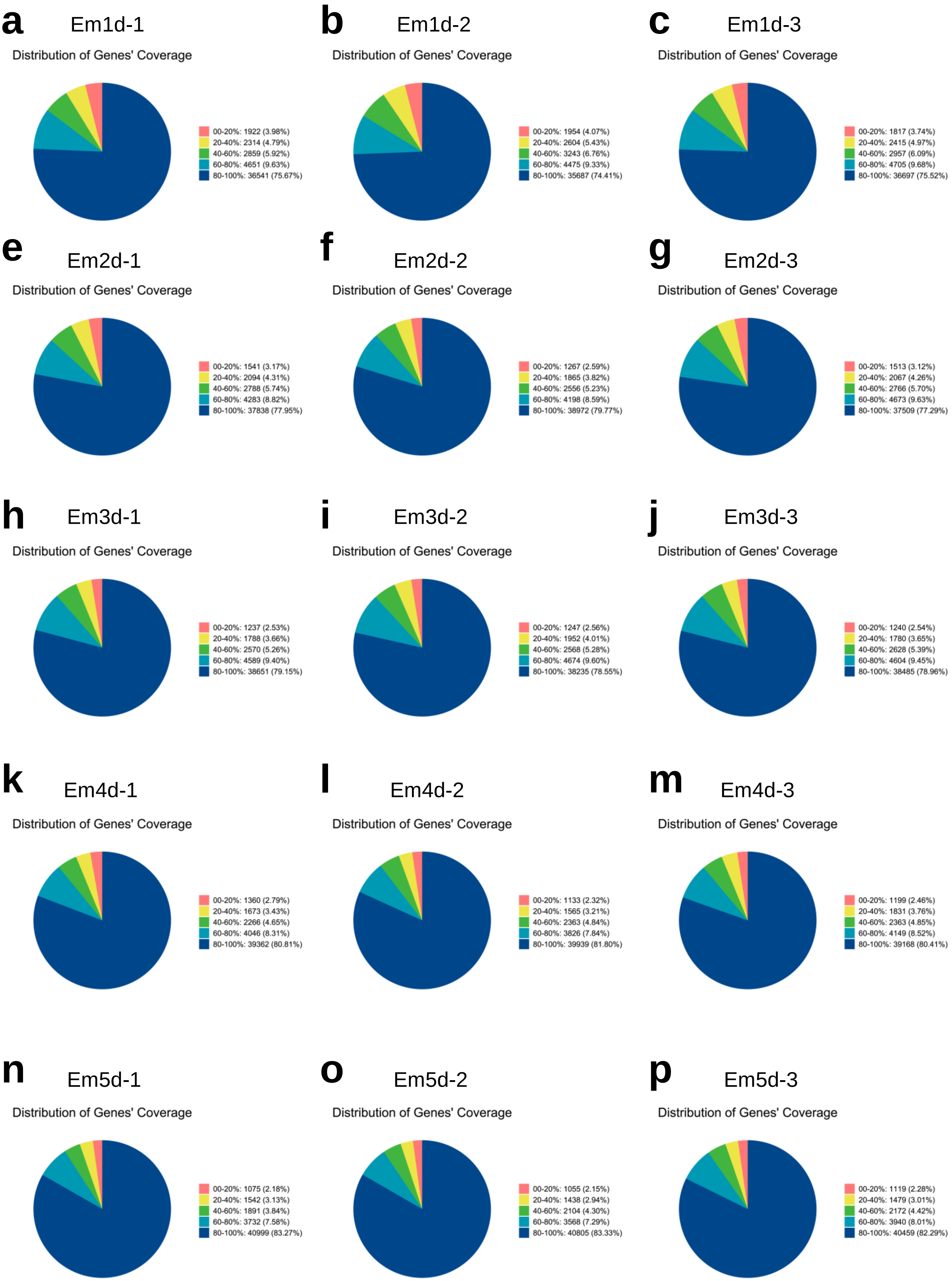

Figure S2. Gene coverage of different samples. Sample gene coverage distribution.

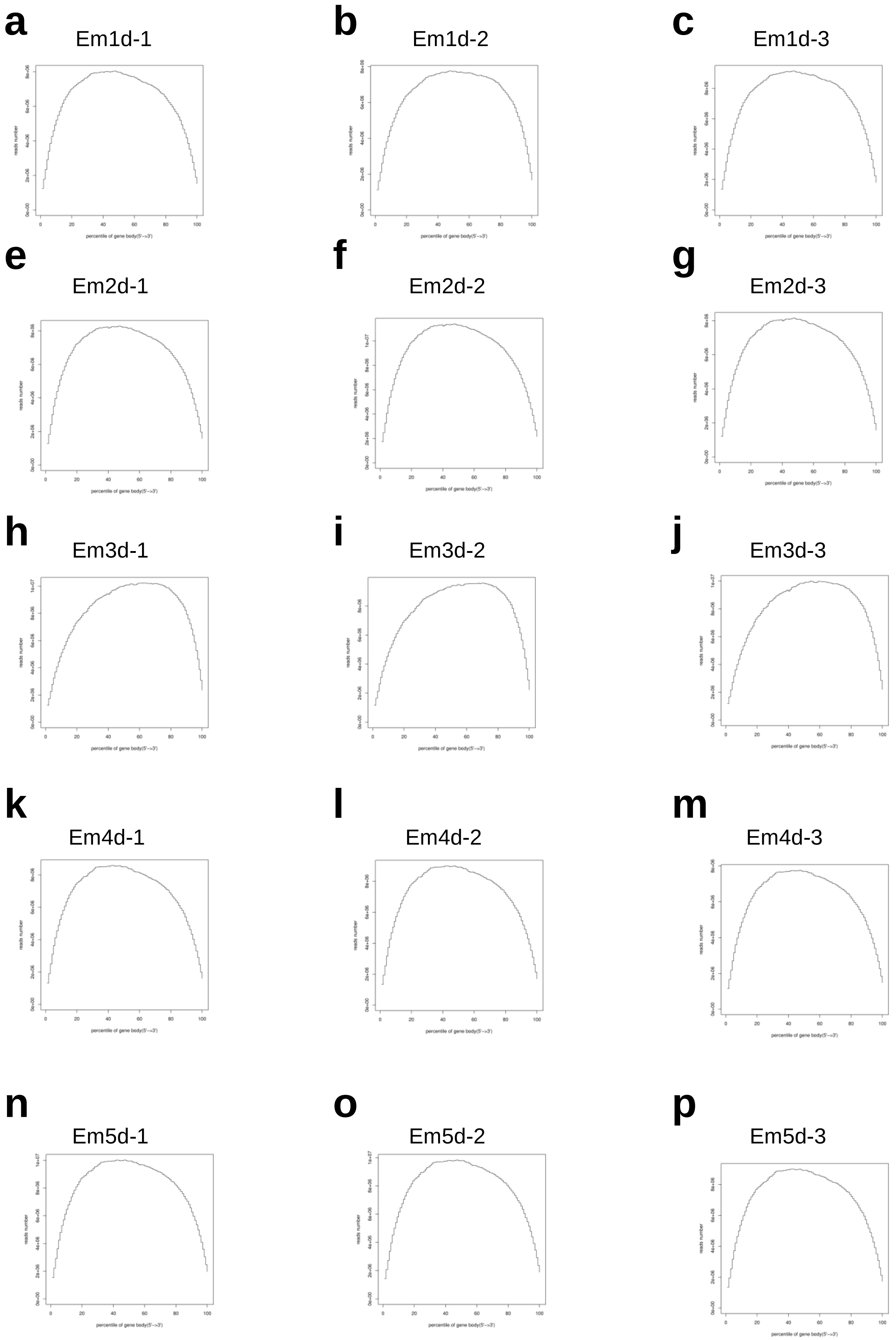

Figure S3. Sample randomness distribution. Random distribution of samples in different periods.
