# Supplementary material for: Transcriptomic analysis reveals the dynamic changes of transcription factors during early development of chicken embryo
Source: BMC Genomics. 2022 Dec 13;23:825. doi: 10.1186/s12864-022-09054-x (PMC9746114; doi:10.1186/s12864-022-09054-x)
Supplement: Supplementary file 2 — Additional file 2: Table S1. Data quality assessment in sequencing. [file 12864_2022_9054_MOESM2_ESM.docx]

**Supplementary Information**

Supplementary file 1: **Table S1.** Data quality assessment in sequencing. **Table S2.** Distribution of TFs in genome. **Table S3.** Common TFs highly expressed in all stages. **Table S4.** Go enrichment of DE-TF in different stages. **Table S5.** Differentially expressed transcription factors (DE-TFs). **Figure S1.** Overview of RNA-seq mapping in chicken genome. **Figure S2.** Gene coverage of different samples. **Figure S3.** Sample randomness distribution.
